# RETRACTION NOTICE: Tripterine up-regulates miR-223 to alleviate lipopolysaccharide-induced damage in murine chondrogenic ATDC5 cells

**DOI:** 10.1177/20587384211040396

**Published:** 2021-09-02

At the request of the Journal Editor(s) the following article has been retracted.

Li X, Wei W, Zhao Z, Lv S (2019) Tripterine up-regulates miR-223 to alleviate lipopolysaccharide-induced damage in murine chondrogenic ATDC5 cells. *International Journal of Immunopathology and Pharmacology* 33: 1-11. DOI: 10.1177/2058738418824521.

Significant similarities between this paper and a number of other papers have been identified, as well as indication of image and data manipulation suggestive of the use of a “paper mill”. These papers share common structures and wording yet do not share any common authors.

The authors did not provide a sufficient response or evidence to refute these claims. As a result, the scientific accuracy of this paper is not reliable.

Adhering to the international guidelines established by the Committee on Publication Ethics, the Journal has determined these are grounds for retraction.

